# The role of P3H family in cancer: implications for prognosis, tumor microenvironment and drug sensitivity

**DOI:** 10.3389/fonc.2024.1374696

**Published:** 2024-04-19

**Authors:** Ziyun Wang, Hua Wang

**Affiliations:** Department of Breast and Thyroid Surgery, Affiliated Hospital of Nantong University, Medical School of Nantong University, Nantong, China

**Keywords:** P3H family, tumor microenvironment, immune infiltration, drug resistance, precision medicine

## Abstract

**Introduction:**

Prolyl 3-hydroxylases (P3H) are crucial enzymes in collagen biosynthesis and are known to be involved in a variety of physiological processes. However, their specific roles in cancer progression, modulation of the tumor microenvironment (TME), and impact on patient prognosis remain areas that require further investigation.

**Methods:**

The investigation involved a comprehensive analysis of expression profiles and clinical data obtained from the Genotype-Tissue Expression (GTEx) and The Cancer Genome Atlas (TCGA) databases. This included the assessment of genetic variation, gene expression, and the prognostic significance of P3H family genes. P3H scores were calculated using various databases and R-based tools, followed by correlation analyses with the TME, immune cell infiltration, drug sensitivity and immunotherapy.Variations in P3H gene expression patterns were observed across different tumor types and prognoses, suggesting that most genes within the family were risk factors, especially P3H1 and P3H4. The P3H score was associated with immune infiltration and drug resistance. Notably, individuals with elevated expression of P3H2, P3H3, and CRTAP exhibited higher resistance to multiple anti-tumor drugs.

**Results:**

P3H family proteins play diverse roles in cancer progression, significantly impacting patient prognosis and the effectiveness of immunotherapy.

**Conclusions:**

The P3H score, identified as a potential biomarker for evaluating TME, holds promise in guiding precision medicine strategies.

## 1 Introduction

The extracellular matrix (ECM) is a complex network of proteins and polysaccharides crucial for preserving tissue structure and facilitating cell communication. Collagen, the predominant protein in the ECM, is vital for maintaining tissue rigidity and modulating signaling pathways that influence cell behaviors such as growth, migration, and differentiation (1). Alterations in collagen expression and modifications are frequently observed in various cancers, impacting the remodeling of the tumor microenvironment (TME), disease progression, and patient prognosis (2).

The Proline 3-hydroxylase (P3H) family, which includes P3H1, P3H2, P3H3, P3H4, and CRTAP, has garnered attention in cancer research due to its role in hydroxylating proline residues in collagen. This enzymatic family is crucial for the proper folding and stability of collagen’s triple helix structure (3). In the context of tumor development, P3H enzymes influence the biomechanical properties of collagen, impacting the tumor microenvironment (TME) (4). Studies have linked differential expression of P3H family members to cancer prognosis, such as the association of P3H1 with various tumors and elevated P3H2 and P3H3 levels in aggressive breast cancer (5). Further exploration of their involvement in cancer can shed light on tumor invasion, metastasis, and interactions with the tumor stroma (6). The TME, comprising immune cells, fibroblasts, blood vessels, and the extracellular matrix (ECM), plays a critical role in cancer progression. Collagen within the ECM modulates immune cell recruitment and function, potentially influencing the efficacy of immunotherapy (7). Understanding how P3H enzymes affect collagen maturation in the TME may unveil novel strategies to manipulate immunosuppression in tumor regions and impact immunotherapy outcomes (8). Additionally, research has delved into the P3H family’s role in drug sensitivity, particularly in relation to collagen’s structural integrity and its implications for drug delivery within the TME (9). Enzymes involved in collagen maturation, like P3H, could alter the mechanical properties of the TME, affecting cancer cell responses to treatment (10).

We examined P3H family genes across 33 tumor types, analyzing their expression patterns, clinical characteristics, prognostic associations, treatment responses, and genetic changes including mutations and copy number variations. We calculated the P3H score and assessed its correlation with the tumor immune microenvironment (TIME) to predict the potential efficacy of immunotherapy.

## 2 Methods

### 2.1 Data acquisition

Expression profiles and clinical information for Genotype-Tissue Expression (GTEx) and The Cancer Genome Atlas (TCGA) were obtained from the University of California, Santa Cruz (UCSC) Xena database (11)(https://xenabrowser.net/datapages/). The abundance of immune cells within the TCGA database was elucidated using the Immune cell abundance identifier (ImmuCellAI) (12)(http://bioinfo.life.hust.edu.cn/ImmuCellAI#!/)and the TIMER2 database (13)(http://timer. cistrome.org/), providing insights into immune cell infiltration levels. The immunotherapy data sets GSE13507 and GSE91061 were downloaded from the Gene Expression Omnibus (GEO) database (https://www.ncbi.nlm.nih.gov).

### 2.2 Genomic analysis

Genomic modifications within the P3H family, including mutations, copy number variations (CNVs), and methylation patterns, were examined using the Gene Set Cancer Analysis (GSCA) database, available at http://bioinfo.life.hust.edu.cn/GSCA/#/. Spearman analysis was performed to determine the strength of the association.

### 2.3 P3H score analysis

The P3H score for each patient in the TCGA cohort was calculated using the single-sample gene set enrichment analysis (ssGSEA) function within the ‘GSVA’ R package (version 4.2.2).In this research, we utilized the ssGSEA function within the R package ‘GSVA’ to assess the activity of the P3H family gene set in pan-cancer samples. The approach involved sorting the gene expression levels in each sample, followed by filtering and aggregating the gene expression levels within the gene set. By performing 1000 random rearrangements and enrichment score calculations, we utilized the empirical cumulative distribution function (ECDF) to determine the P3H score for each sample, reflecting the gene set’s activity. This method offers a succinct measure of P3H activity in each sample, enabling comparative analysis across different cancer types.

### 2.4 P3H score prognostic analysis

The impact of the P3H score on patient survival outcomes across various cancer types was assessed through univariate Cox regression (uniCox) analysis. Overall survival (OS), disease-specific survival (DSS), disease-free interval (DFI), and progression-free interval (PFI) were analyzed using the ‘survminer’ and ‘survival’ libraries in the R programming environment. The impact of P3H score on the prognosis of patients with different types of cancers was evaluated using the Cox proportional hazard model. Hazard ratio (HR) within the 95% confidence interval and corresponding P values were calculated. These findings were visualized in heat maps using the ‘ggplot2’ package in R. Additionally, Kaplan-Meier survival analysis was employed to investigate the prognostic relevance of P3H score across various human cancer types.

### 2.5 Analysis of gene enrichment

Gene set variation analysis (GSVA) enrichment analysis was performed to explore the biological functions of the P3H group and its associations with various cancer types, utilizing the ‘GSVA’ package in R. The correlation between P3H scores and the 50 HALLMARK pathways was analyzed using the Molecular Signatures Database (MsigDB), accessible at http://software.broadinstitute.org/gsea/msigdb/index.jsp.

### 2.6 Tumor microenvironment analysis

The ‘ESTIMATE’ R package was employed to determine the stromal score, immunological score, and tumor purity score for each patient in the TCGA cohort. Correlations between these scores and P3H scores were examined. Additionally, correlations between P3H scores and various elements, including the degree of immune cell infiltration, expression of immune regulatory genes, and genes associated with TGFB1 and epithelial-mesenchymal transition (EMT) pathways across different cancer types, were analyzed using the ‘ggplot2’ R package to visualize heatmaps.

### 2.7 Drug sensitivity analysis

The link between the P3H gene family and the efficacy of small molecule therapeutics was investigated using data from the Genomics of Drug Sensitivity in Cancer (GDSC- https://www.sanger.ac.uk/tool/gdsc-genomics-drug-sensitivity-cancer/) and the Cancer Therapeutics Response Portal (CTRP-https://portals.broadinstitute.org/ctrp/). The Pearson correlation coefficient was computed for P3H family proteins and the percentage of drug sensitivity.

### 2.8 Clinical samples

This study examined 57 patients with breast cancer, bladder cancer, and liver cancer who underwent surgery at the Affiliated Hospital of Nantong University in Jiangsu Province from August 2021 to August 2022. Patients included had not undergone radiotherapy or chemotherapy prior to surgery. The study was approved by the Ethics Committee of Nantong University Hospital (Protocol Code 2020-L125), and informed consent was obtained from all participants.

### 2.9 Real-time polymerase chain reaction

Total RNA was extracted from BC and adjacent non-tumor tissues using TRizol (Invitrogen, Life Technologies, Paisley, UK). The integrity and quality of RNA were assessed using a spectrophotometer (Thermo Fisher, USA). Subsequently, the RNA was reverse transcribed into cDNA using the PrimeScript RT kit (Takara, Dalian, Liaoning, China). Repeated RT-PCR studies were performed on a LightCycler 480 instrument (Roche, Switzerland), and QRT-PCR was carried out using SYBRGreenIMastermix. The mRNA expression levels of the gene of interest were normalized by ACTIN and quantified using the 2-ΔΔCT technique. The primer sequences used for β-ACTIN were as follows: forward: TAGTTGCGTTACACCCTTTCTTG, reverse: GCTGTCACCTTCACCGTTCC. The primer sequences for P3H1 were: forward: GGCAGCAACCTCAGGAGATGG, reverse: AGGGCTTTGAAGACAGTGACACC. All primers were obtained from Shanghai Sangon Technology Co., Ltd. The primer sequences for P3H4 were: forward: 5’-TCTACCCGGCCATAGCAGATC-3’, reverse: 5’-TTGTCCACGAAGTAGCCACCC-3’.

### 2.10 Immunochemistry

Five cases of breast cancer (BC) and five cases of liver cancer (LIHC) were obtained, along with corresponding paracancerous samples, from the Affiliated Hospital of Nantong University for immunohistochemistry. All patients were pathologically diagnosed with BC or LIHC, and clinicopathological data such as sex and age were collected. The study was conducted in accordance with the Declaration of Helsinki and approved by the Ethics Committee of Nantong University Hospital in China. Antibodies were purchased from the Abcam website in Cambridge, MA, and immunohistochemical staining methods followed the manufacturer’s instructions. Ten fields of view were randomly selected (scale bar = 25 μm), and images were independently read by two researchers. Hematoxylin stains cell nuclei blue.

### 2.11 Statistical analysis

All data were presented as mean ± SD. Student’s t-test was used to analyze and compare variations among groups. Statistical analysis was conducted using R version 4.2.2.(https://www.r-project.org/) Histograms were generated using the ‘ggplot2’ and ‘ggpubr’ R packages. Pearson’s correlation coefficient was employed for correlation analyses. Statistical significance was determined by a significance level of P < 0.05, where *P < 0.05, **P < 0.01, ***P < 0.001, and ****P < 0.0001 denoted different levels of significance.

## 3 Results

### 3.1 P3H family mRNA levels and prognostic value

**Figure 1:**
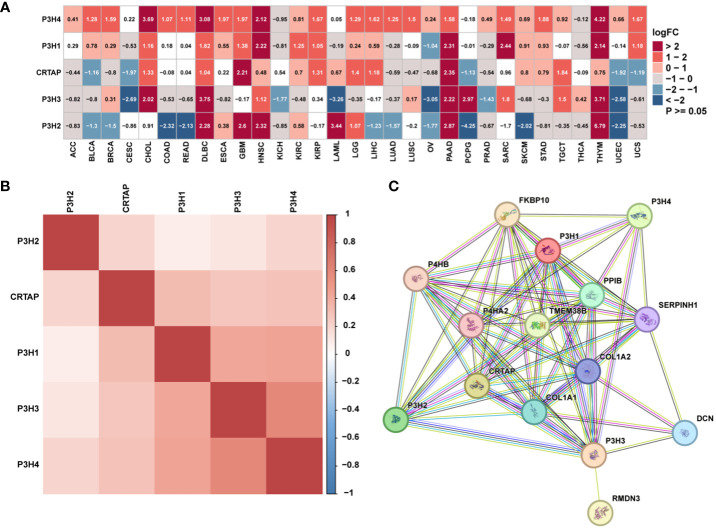
The expression of genes of the P3H family. **(A)** Differential P3H family expression observed in 33 types of tumors using TCGA and GTEx cohorts. **(B)** Correlation between P3H gene family members using pan-cancer data from TCGA. **(C)** The protein-protein interaction network of P3H pathway genes.

**Figure 2:**
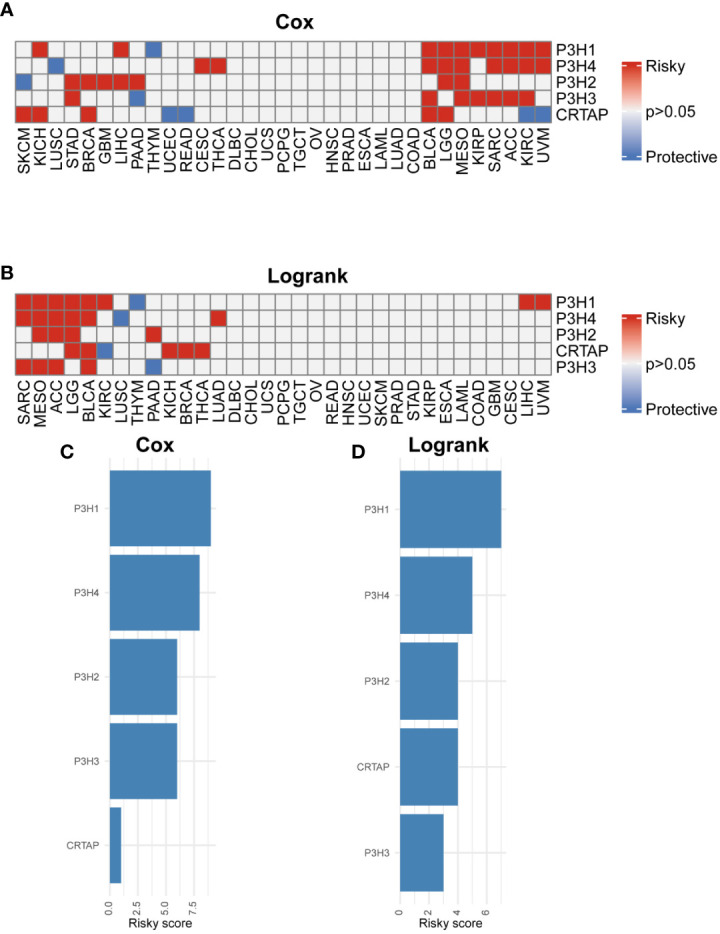
Prognostic value of P3H family genes. **(A, B)** Heat map of Cox results and Logrank results for P3H family genes in each tumor type. **(C, D)** Risk scores of each P3H family gene in pan-cancer.

The research analyzed the expression patterns for five P3H family genes across thirty-three unique tumor varieties, drawing upon data collected from the TCGA and GTEx databases. **Supplementary Table 1** lists abbreviations for 33 cancer types. As shown in **Figure 1A**, the expression of the five P3H family genes varied among the 33 tumor types. We further examined the correlation between P3H family members and the 33 cancer cases (**Figure 1B**), as well as the protein-protein interaction network of P3H pathway genes (**Figure 1C**). Furthermore, we conducted uniCox analysis on every individual gene within the 33 tumors (**Figures 2A, B**). Taken together, our results suggest that the majority of genes belonging to the P3H family are linked to a higher susceptibility to tumors. We calculated a risk score by subtracting the number of tumors with a gene that acts as a protective factor from the number of tumors with a gene that acts as a risk factor. Importantly, *P3H1 and P3H4* was recognized as the foremost determinant of risk (**Figures 2C, D**). PCR results show that P3H1 and P3H4 are differentially expressed in breast cancer, bladder cancer and liver cancer. (**Supplementary Figure 1**) Moreover, high expression of *P3H* correlated with poor overall survival in several tumor types, including KICH, LIHC, BLCA, LGG, MESO, KIRP, SARC, ACC,UVM, and KIRC.

### 3.2 Genetic alterations in the P3H family

**Figure 3:**
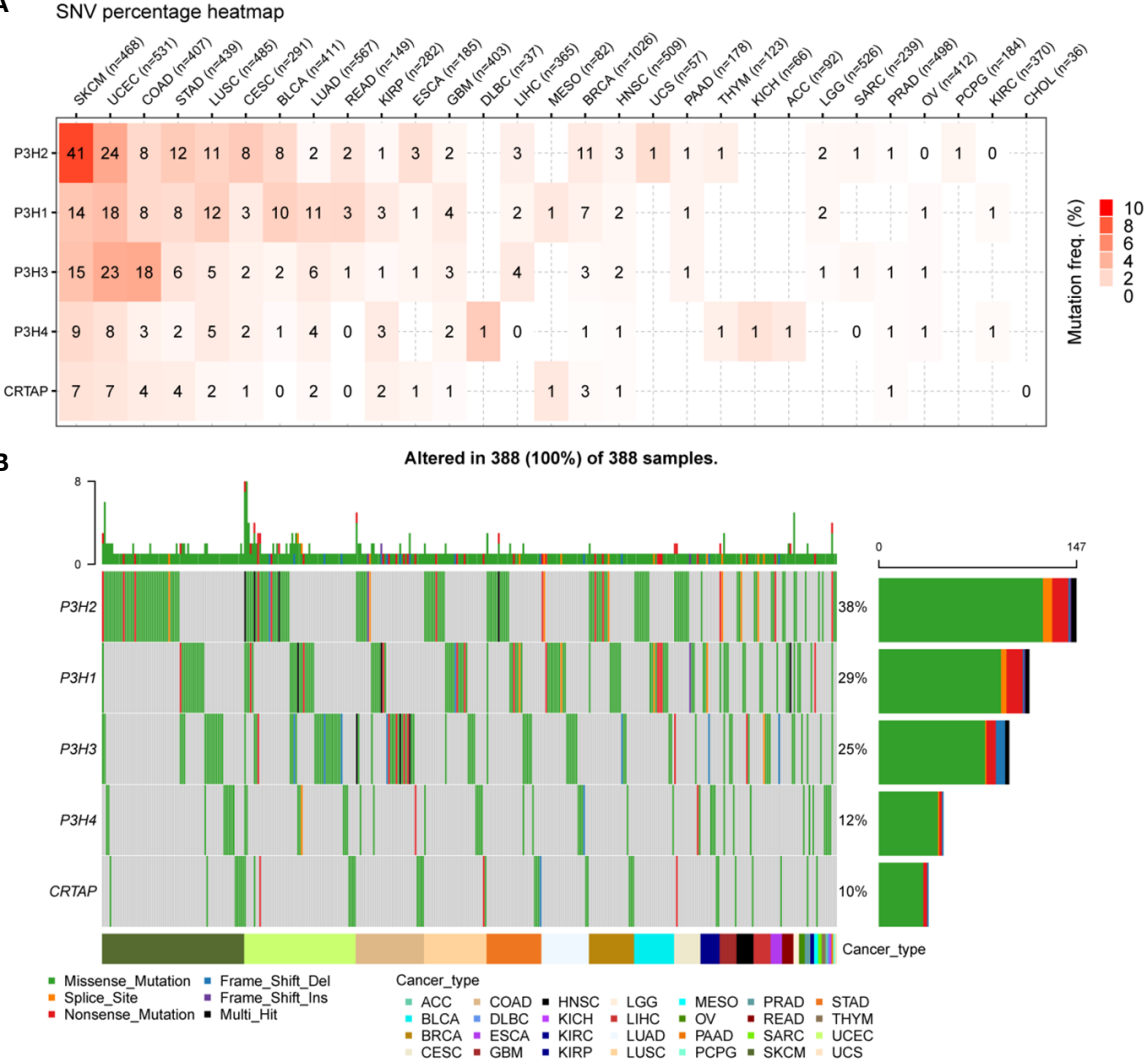
SNV changes in the P3H gene family in all cancer types. **(A)** Overview depicting the occurrence of harmful genetic changes in specific forms of cancer. **(B)** Oncoplot illustrating the distribution of gene mutations in the selected cancers’ sample set based on the inputted gene set.

4Analysis of single nucleotide variants (SNVs) revealed that P3H2 harbored the most deleterious mutations in the context of cutaneous melanoma (SKCM), as illustrated in **Figure 3A**. Additionally, when adopting a pan-cancer perspective, P3H2 exhibited the highest mutation frequency at 38%, relative to its counterparts within the *P3H* gene family, as depicted in **Figure 3B**.

An additional analysis was conducted on the P3H gene family to investigate copy number variations (CNV).

**Figure 4:**
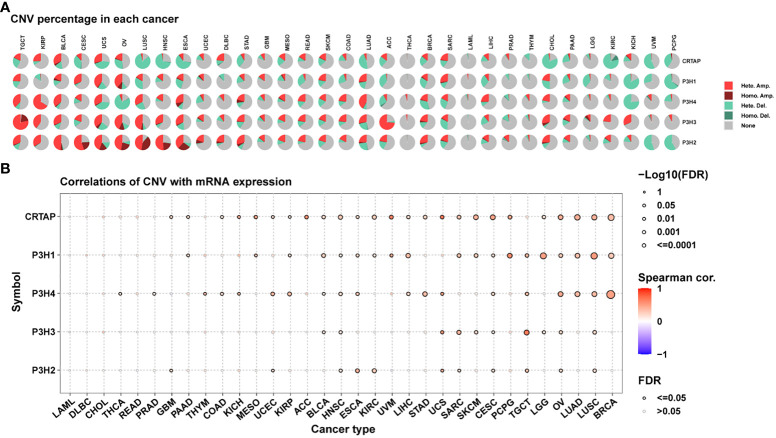
Copy Number Variations (CNVs) of the P3H Family in Pan-Cancer. **(A)** Pie chart illustrating the distribution of CNVs among the P3H family genes in the identified tumor types. **(B)** Exploration of the relationship between gene expression and CNV.

**Figure 4A** presents the distribution of various CNV types, including amplifications and deletions in both heterozygous and homozygous states, across multiple cancer types. It is noteworthy that CRTAP was significantly relevant in 25 out of 33 tumor types, as illustrated in **Figure 4B**.

### 3.3 Estimation, differential distribution, and survival analysis of P3H scores

**Figure 5:**
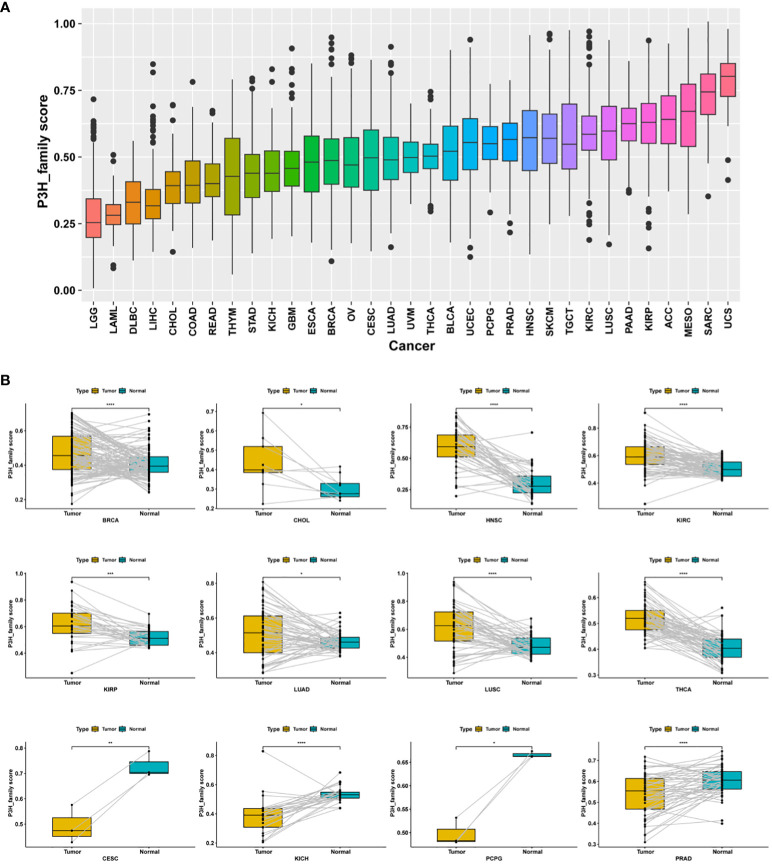
Distribution of differences in P3H scores. **(A)** The TCGA cohort illustrates the distribution of P3H scores among 33 different tumor types. **(B)** Differential distribution of P3H scores in pairs of tumors and adjacent normal tissue. *P < 0.05, **P < 0.01, ***P < 0.001, ****P < 0.0001.

P3H scores were computed across 33 distinct tumor types in the TCGA cohort using ssGSEA. Among these, uterine carcinosarcoma (UCS) exhibited the highest P3H score, while lower-grade glioma (LGG) had the lowest (**Figure 5A**). Notably, P3H scores were significantly elevated in tumor tissues compared to adjacent normal tissues in BRCA, CHOL, HNSC, KIRC, KIRP, LUAD, LUSC, and THCA (as depicted in **Figure 5B**). Results from the univariate Cox analysis revealed several significant associations: (1) The P3H score was linked to an increased.

**Figure 6:**
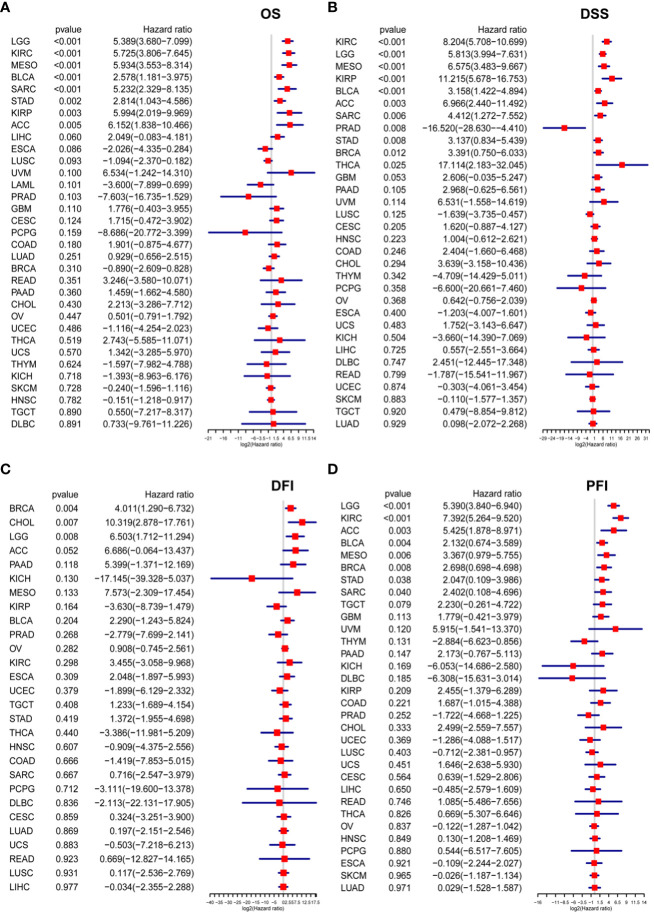
Analysis of P3H scores for survival. **(A, D)** The P3H score emerges from the Cox analysis in a forest plot for pan-cancer patients. **(A)** Overall survival; **(B)** disease-specific survival; **(C)** disease-free interval; and **(D)** progression-free interval.

OS in LGG, KIRC, MESO, KIRP, BLCA, ACC, SARC, and STAD as depicted in **Figure 6A**; (2) Regarding DSS, the P3H score emerged as a risk factor for LGG, KIRC, MESO, KIRP, BLCA, ACC, SARC, STAD, BRAC, and THCA, while it presented a protective effect for PRAD, as illustrated in **Figure 6B**; (3) The P3H score was recognized as a prognostic risk factor impacting the DFI for CHOL and LGG. (**Figure 6C**); (4) Regarding PFI, the P3H score was found to be a risk factor for LGG, KIRC, ACC, BLCA, MESO, BRCA, STAD, and SARC (**Figure 6D**).

### 3.4 GSVA of P3H score

**Figure 7:**
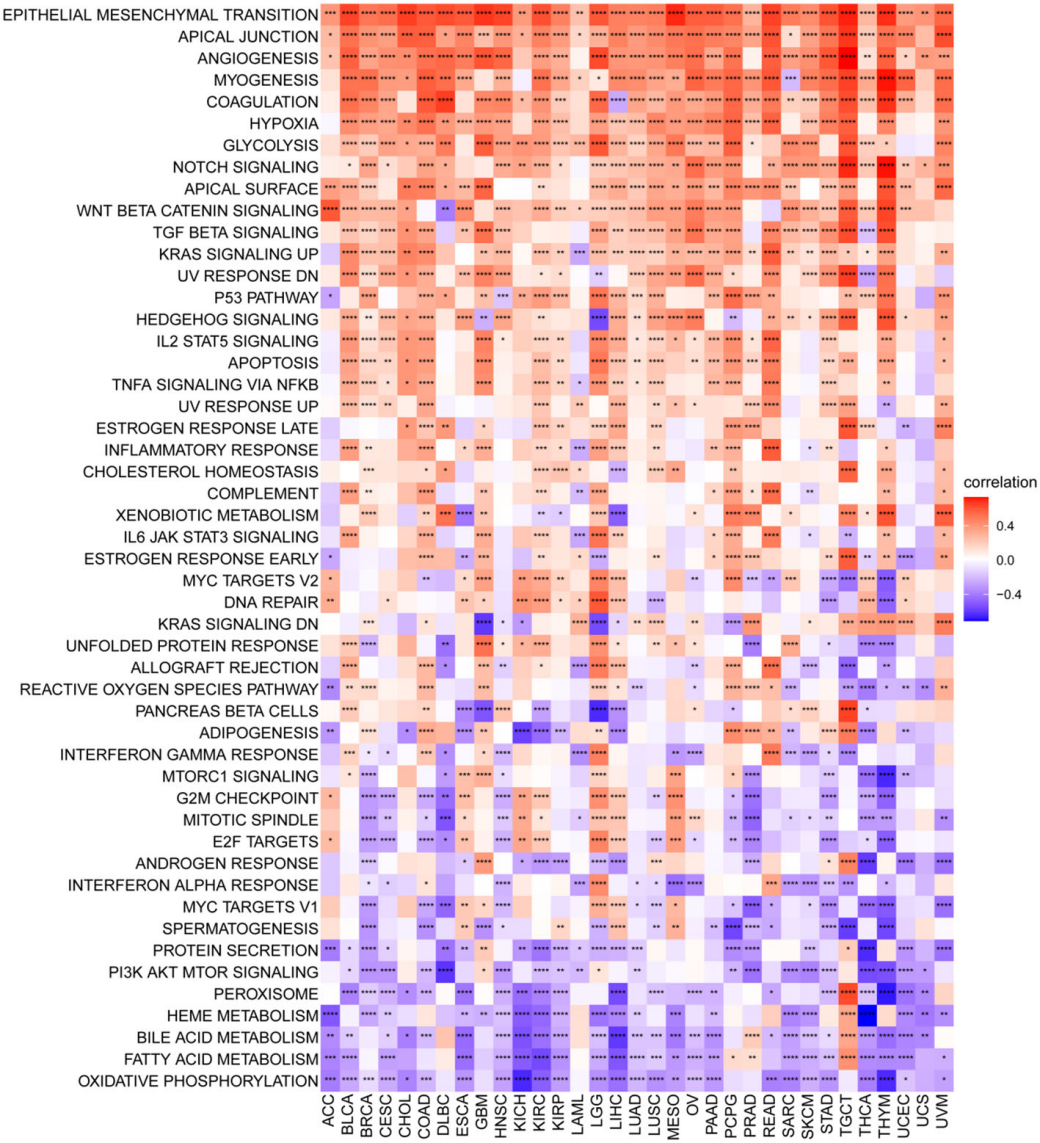
GSVA for the P3H grade. Pan-cancer heatmap showing the relationship between 50 Hallmark pathways and P3H score. *P < 0.05, **P < 0.01, ***P < 0.001, ****P < 0.0001.

To elucidate the potential pathways influenced by the P3H score, we performed an analysis of 50 HALLMARK pathways utilizing GSVA. **Figure 7** illustrates the correlation between the P3H score and GSVA score in pan-cancer. Our study results suggest a robust association between the P3H score and various cancerous pathways in pan-cancer, including IL6-JAK-STAT3 signaling, IL2 STAT5 SIGNALING response, and the inflammatory response. Notably, these pathways exhibit a strong connection with the Tumor Immune Microenvironment.

### 3.5 Relationship between P3H scores and TME

**Figure 8:**
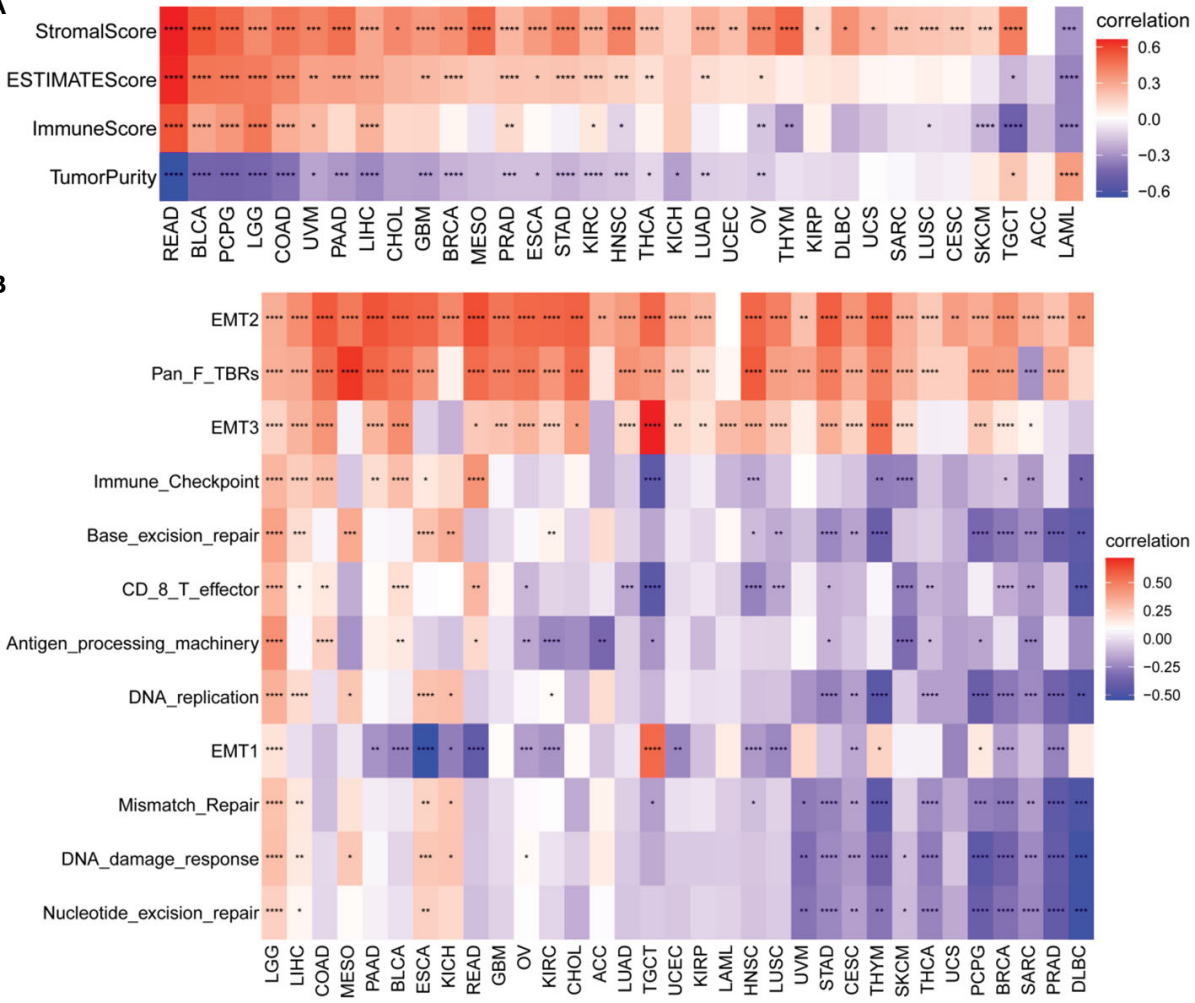
TME analysis of P3H scores. **(A)** Heatmap of the correlation of P3H score with the pan-cancer immune score, stromal score, ESTIMATE score, and tumor purity score. **(B)** Heatmap of correlation of P3H scores with TME-related pathways. *P < 0.05, **P < 0.01, ***P < 0.001, ****P < 0.0001.

We also identified a positive correlation between the P3H score and the immunological, stromal, and ESTIMATE scores in the majority of malignancies (**Figure 8A**). To gain further insights, we conducted an extensive examination of TME-associated pathways, with a particular emphasis on pathways related to the immune system, extracellular matrix, and DNA repair, as documented in existing literature. Our findings revealed a robust association between the P3H score and matrix-related pathways, such as EMT2, EMT3, and Pan_F_TBRs (**Figure 8B**).

### 3.6 Immune infiltration analysis

**Figure 9:**
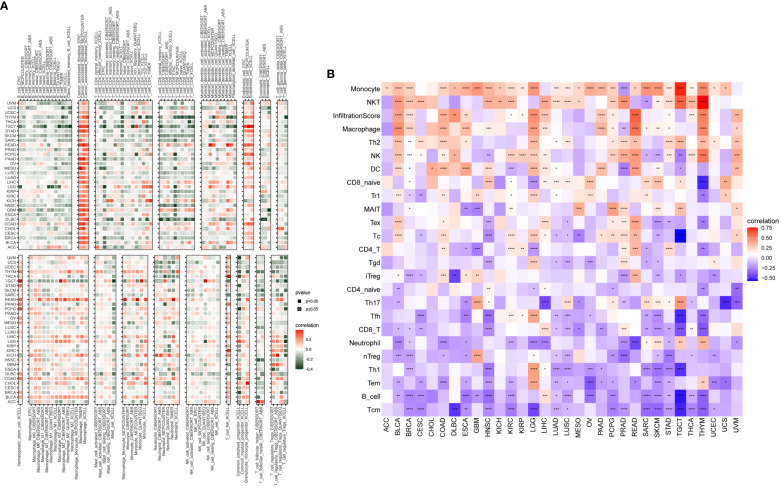
An analysis of immunological infiltration. Assessment of how the P3H score correlates with immune cell infiltration was performed by utilizing **(A)** the TIMER2 database and **(B)** the ImmuCellAI tool for analysis. *P < 0.05, **P < 0.01, ***P < 0.001, ****P < 0.0001.

In our study, we explored the relationship between P3H scores and immune-related cells in the TME. Through the examination of data from the TIMER2 (**Figure 9A**) and ImmuCellAI databases, we identified a robust association between the P3H score and the infiltration of immune cells across various types of cancer. Notably, the strongest connection between the P3H score and tumor-associated fibroblasts indicated an immune-activated TME (**Figure 9B**).

**Figure 10:**
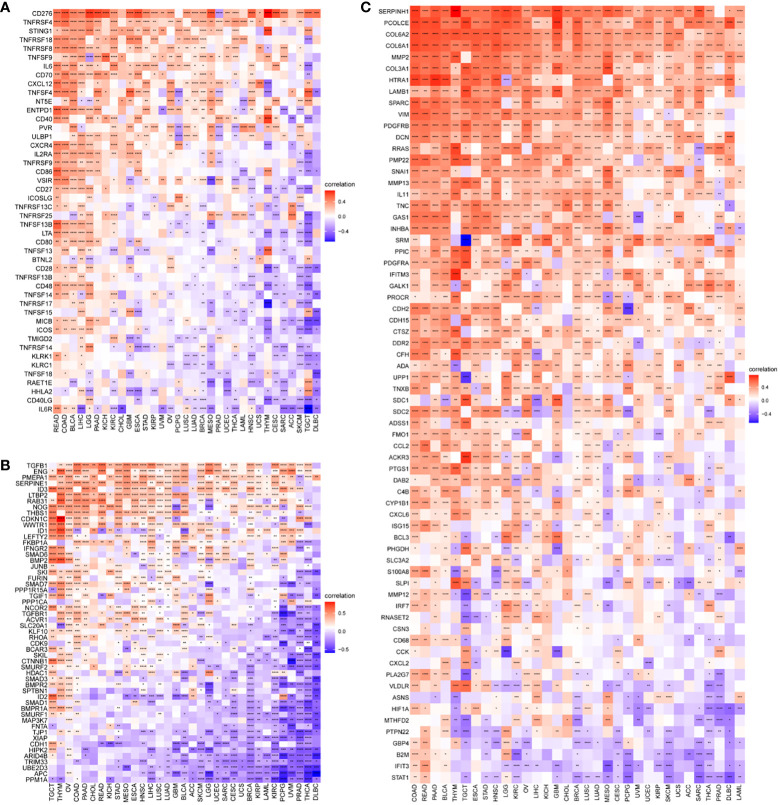
Correlation between P3H scores and immune-related genes. **(A)** Immunosuppressive gene, immune checkpoint **(B)** TGFB1 pathway gene **(C)** EMT pathway gene. *P < 0.05, **P < 0.01, ***P < 0.001, ****P < 0.0001.

The results of our study indicate a correlation between P3H scores and immune suppression-related genes, immune checkpoint genes (**Figure 10A**), TGFB1 pathway-related genes (**Figure 10B**), and EMT pathway-related genes (**Figure 10C**) in most types of tumors. Furthermore, higher P3H scores in patients are associated with increased infiltration of immune cells, suggesting potential for more favorable responses to immunotherapeutic treatments.

### 3.7 Association between P3H score and immunotherapy response

**Figure 11:**
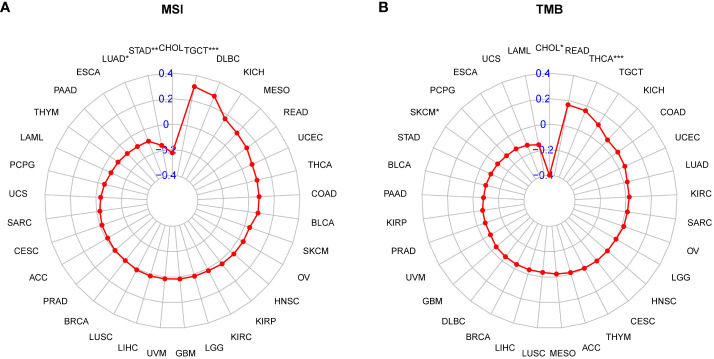
Correlation between P3H scores and MSI and TMB values. **(A, B)** Radar plots of correlations between P3H scores and **(A)** MSI or **(B)** TMB.

**Figure 12:**
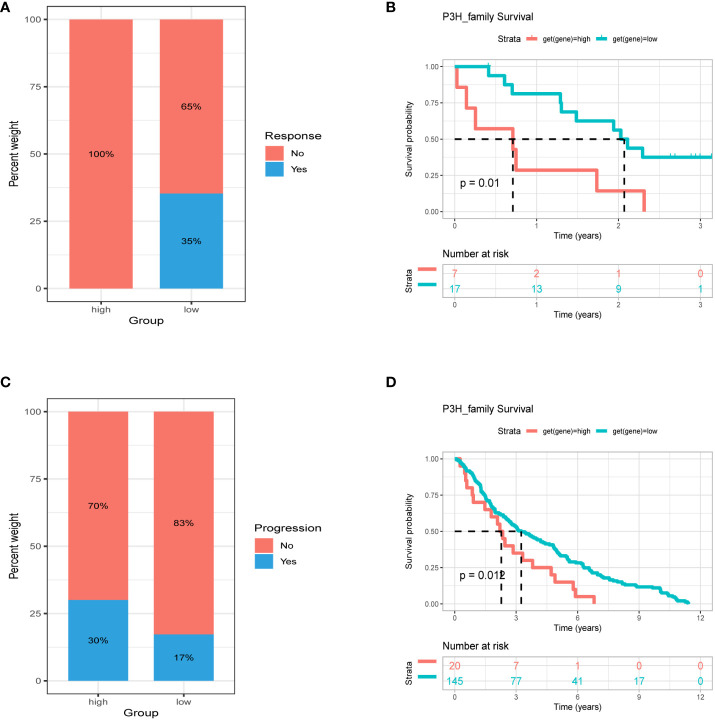
Association between P3H score and immunotherapy response. Percentage of patients with progression in the high and low P3H score groups in the **(A)** ICB.Riaz2017_Nivolumab_Melanoma_Naive and **(C)** GSE13507 cohorts. **(B)** OS analysis by P3H score in the ICB.Riaz2017_Nivolumab_Melanoma_Naive cohort. **(D)** OS analysis by P3H score in the GSE13507 cohort. OS, overall survival.

Patients with high tumor mutation burden (TMB) or microsatellite instability (MSI) may exhibit sensitivity to immunotherapy. Our study revealed that the P3H score was linked to MSI in three cancer types and TMB in three cancer types. Specifically, P3H scores in LUAD and STAD showed a negative correlation with MSI values (**Figure 11A**), while P3H scores in SKCM and CHOL displayed a negative correlation with TMB values (**Figure 11B**). These findings suggest that patients with low P3H scores may respond well to immunotherapy. To investigate this further, we analyzed immunotherapy data alongside P3H scores. Our Kaplan-Meier analysis indicated that patients with low P3H scores who underwent ICI therapy had improved overall survival (OS) or progression-free survival (PFS) rates. Conversely, a higher proportion of patients in the high P3H score group experienced disease progression (**Figures 12A–D**).

### 3.8 Drug sensitivity correlation analysis

**Figure 13:**
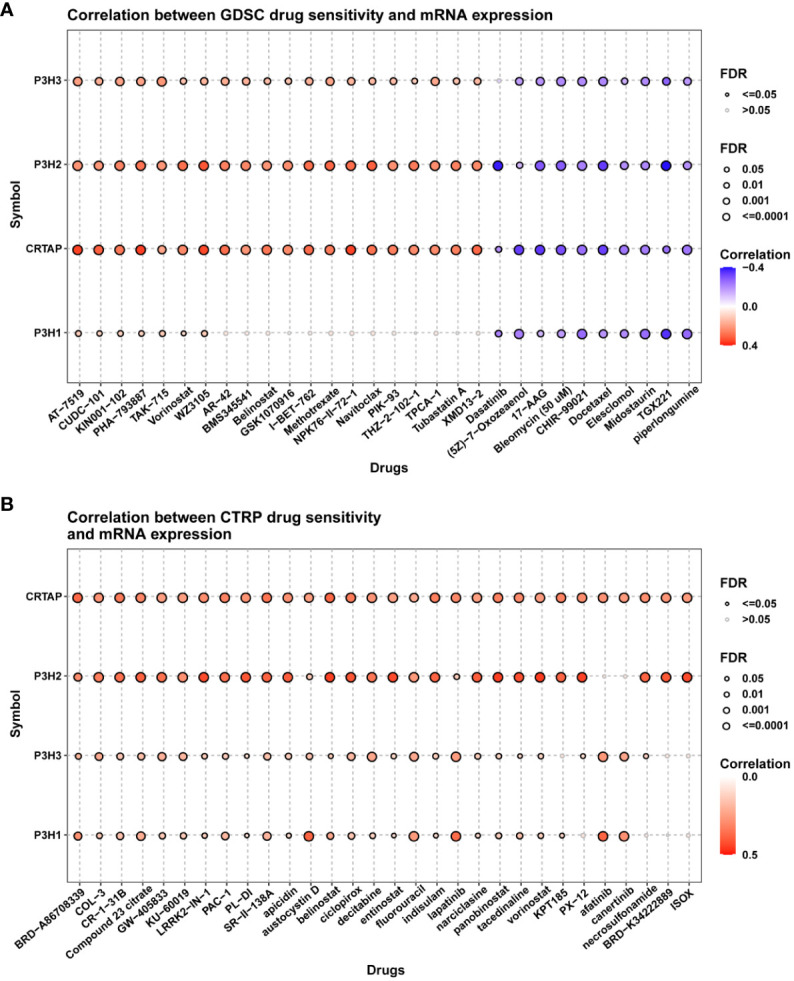
A correlation analysis between drug sensitivity and the expression of P3H family genes. The GDSC **(A)** and CP3H **(B)** datasets are used in the analysis.

To investigate the involvement of P3Hs in chemotherapy, we conducted a correlation analysis to explore the connection between the expression of P3Hs and drug sensitivity. Our findings, illustrated in **Figure 13**, indicate that high expression levels of P3Hs, particularly *P3H3*, *P3H2*, and *CRTAP*, were correlated with increased resistance to multiple anti-tumor drugs.

### 3.9 Immunohistochemistry

**Figure 14:**
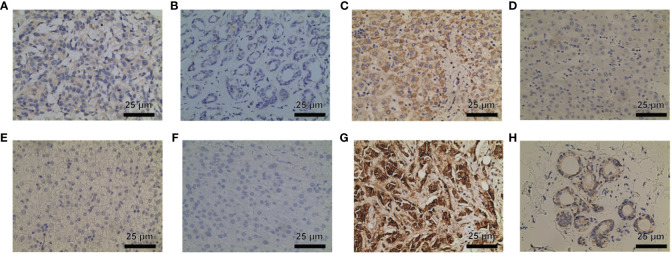
P3H1 is in breast cancer tissue **(A)** and paracancerous tissue **(B)**; CRTAP is in liver cancer tissue **(C)** and paracancerous tissue **(D)**; P3H1 is in breast cancer tissue **(E)** and paracancerous tissue **(F)**; P3H3 is in breast cancer tissue **(C)** and paracancerous tissue **(D)** Immunohistochemistry results of cancer tissue **(G)** and paracancerous tissue **(H)**. Hematoxylin stains the cell nucleus blue, and DAB positive expression appears brown. DAB, diaminobenzidine.

In order to further validate the findings on P3H family protein expression levels, immunohistochemical staining was conducted. The results indicated elevated levels of P3H1/3 in BC tissue compared to adjacent normal tissue, and higher levels of P3H1 and CRTAP in LIHC tissue compared to adjacent normal tissue. (**Figures 14A–H**).

## 4 Discussion

Tumor development relies heavily on the intricate composition of the TME, encompassing a diverse array of cellular and non-cellular components. Collagen, the primary structural protein of the TME’s key non-cellular component, the Extracellular Matrix (ECM), plays a crucial role in tumor growth, invasion, and spread The tightness and rigidity of collagen are regulated by different modifying enzymes, including the P3H family, which hydroxylates the proline residues of collagen (6). This hydroxylation process significantly affects collagen cross-linking and the mechanical properties of specific tissues (14, 15). However, limited research has been conducted on P3H’s role in malignancies or its potential as a therapeutic target.

Pan-cancer analysis is a comprehensive research approach that examines various types of cancer simultaneously. This method allows for the identification of both commonalities and differences among different cancer types, aiding in the understanding of cancer pathways, molecular mechanisms, and diagnostic and treatment strategies. Previous studies have utilized pan-cancer analysis effectively. For instance, Shen Pan et al. (2022) conducted a study on the RUNX protein family using this method (16), while Jindong Xie et al. (2022) performed a pan-cancer multi-omics analysis on the FOXO family (17). Additionally, Bo Tian et al. (2023) investigated the carcinogenic role of Golgi transporter 1B in human tumors through a pan-cancer analysis study (18).

This research involved an extensive pan-cancer evaluation of the P3H gene family across 33 diverse cancer types. Gene mutations, CNV, expression patterns, and clinical characteristics were examined to assess their associations, prognostic value, and drug sensitivity. To evaluate the connection between these indicators and the TIME, as well as immunotherapy response, a P3H scoring system was established with the aim of accurately predicting immunotherapy response. Earlier research has suggested that P3H1 may act as a cancer-causing gene and serve as a separate indicator of unfavorable prognosis in BRCA. Furthermore, it has been found to improve responsiveness to medications such as docetaxel, cisplatin, vinblastine, camptothecin, and paclitaxel (19). Additionally, P3H1 is closely associated with various tumors, particularly LIHC (20), and plays a role in extracellular matrix remodeling and immune suppression in colorectal cancer cells (21). The transcription of tumor suppressor genes *CMAHP*, *TP63*, and *P3H2 (* 22) is disrupted by HPV-human fusion transcripts that are expressed at high levels. In contrast, based on our findings, we suggest that P3H3 functions as a novel form of cancer inhibitor, as its protein manifestation exhibits an inverse association with lymph node spread and tumor development in lung carcinoma (23). Moreover, P3H2 might be a viable target for the treatment of OSCC (24). The potential of METTL3 as a therapeutic target is highlighted by its ability to affect the proliferation, metastasis, and EMT progression of bladder cancer through the regulation of P3H4 (25).

By analyzing data from the TCGA and GTEx databases, we observed significant variations in the expression of P3H family genes across 33 different types of cancer. The elevated expression of most P3H family genes in cancer samples suggests an increased risk, particularly for *P3H1*, which is associated with poorer overall survival in several cancers. SNV analysis revealed that *P3H2* harbored the highest number of deleterious mutations in SKCM and demonstrated significant mutation rates in pan-cancer analysis. Furthermore, our study identified CNVs associated with P3H family genes, with CRTAP showing a significant association in 25 of the 33 cancer types studied. These genome-specific features are crucial for understanding the role of the P3H family in tumor development and their potential as therapeutic targets.

The P3H scoring system displayed promising potential in distinguishing tumors from adjacent normal tissue, indicating its usefulness as a biomarker. In specific cancer types, the level of the P3H score is closely associated with the survival prognosis of patients. Using GSVA, we found a favorable association between the P3H score and various pathways associated with the advancement of cancer, including those closely linked to the tumor microenvironment. A detailed evaluation of the link between the P3H score and the tumor microenvironment revealed a recurrent and positive association with immunologic, stromal, and ESTIMATE scores in most cancer types. Additionally, the P3H score is associated with key tumor microenvironment processes, such as the EMT and inflammatory responses, providing insights into the interactions between tumor cells and their environment.

The robust correlation observed between the P3H score and immune cell infiltration into the TME, particularly the quantity of cancer-associated fibroblasts (CAFs), suggests that the TME may be in an active state that could significantly impact the effectiveness of immunotherapy. A higher P3H score also correlates significantly with the upregulation of genes involved in immunosuppression, immune checkpoints, and the EMT pathway across different tumor categories, indicating the complex interaction between P3H expression levels and the tumor’s resistance strategy against the immune response. This provides valuable insights for tailoring treatments based on individual patient circumstances. The analysis of drug sensitivity indicated that increased expression of members from the P3H family, particularly P3H3 and P3H2, is strongly associated with heightened resistance to specific therapeutic drugs used for treating tumors, suggesting their role in mechanisms leading to tumor resistance. The effectiveness of immunotherapy is influenced by various factors, and our research suggests that patients with lower P3H scores may have a better response to immunotherapy. This is supported by the correlation between higher tumor mutational burden (TMB) and microsatellite instability (MSI), which could enhance sensitivity to immune checkpoint inhibitors (ICIs). Therefore, the P3H score could potentially be used as a biomarker to predict ICI response, a crucial aspect in cancer treatment strategies (26). Immunohistochemical staining further validated our molecular findings, showing higher expression of P3H1/3 in breast cancer (BC) tissues and increased levels of P3H1 and CRTAP in liver cancer (LIHC) tissues. This visual confirmation of protein expression aligns with our genetic data and suggests that P3H expression might be valuable as a diagnostic or prognostic indicator.

Further investigation is needed to fully understand the intricate impact of the P3H family on cancer biology. While there are indications that members of the P3H family could be potential therapeutic targets or biomarkers for cancer prognosis and treatment response, there are challenges that need to be addressed. The complex effects of P3H family genes on the tumor microenvironment (TME) and immune system need to be carefully examined, especially considering the importance of collagen modification and extracellular matrix (ECM) remodeling in cancer progression (27–30). Additionally, delving into the repercussions of targeting the P3H family on cell signaling within the ECM and TME could offer valuable insights into minimizing the adverse effects of cancer therapies (31).

These findings underscore the pivotal role of the P3H family in tumor biology and its potential clinical applications in patient prognosis and personalized cancer treatment strategies. Further investigation should prioritize understanding the precise impact of P3H family proteins on the composition and operation of collagen within the TME, as well as their involvement in the aggregation of immune cells and interaction with immune checkpoint pathways. A comprehensive understanding of these intricate molecular mechanisms will facilitate the identification of new biomarkers and therapeutic targets, optimize patient selection for existing treatment options, and guide the design and development of novel drugs.

## 5 Conclusions

This study conducted a comprehensive analysis of the role of P3H family proteins in 33 types of cancer, revealing their multifaceted involvement in the tumor microenvironment and tumor progression. Significant relationships were observed between the expression patterns, mutations, and copy number variations of P3H family genes and patient prognosis. The implementation of the P3H scoring scheme played a crucial role in enhancing the understanding of the TME and the response to immunotherapy. The identified findings present potential biomarkers and therapeutic targets for future precision medicine applications.

## Data availability statement

The original contributions presented in the study are included in the article/**Supplementary Material**. Further inquiries can be directed to the corresponding author.

## Ethics statement

The studies involving humans were approved by The Ethics Committee of the Affiliated Hospital of Nantong University (protocol code 2020-L125). The studies were conducted in accordance with the local legislation and institutional requirements. The participants provided their written informed consent to participate in this study.

## Author contributions

ZW: Writing – original draft, Writing – review & editing. HW: Writing – review & editing.

## Supplementary material

Supplementary Figure 1 Validation of clinical samples.

Supplementary Table 1 Abbreviations and sample size for 33 cancer types.
