# Supplementary figures and images for: The role of P3H family in cancer: implications for prognosis, tumor microenvironment and drug sensitivity
Source: Front Oncol. 2024 Apr 19;14:1374696. doi: 10.3389/fonc.2024.1374696 (PMC11066264; doi:10.3389/fonc.2024.1374696)

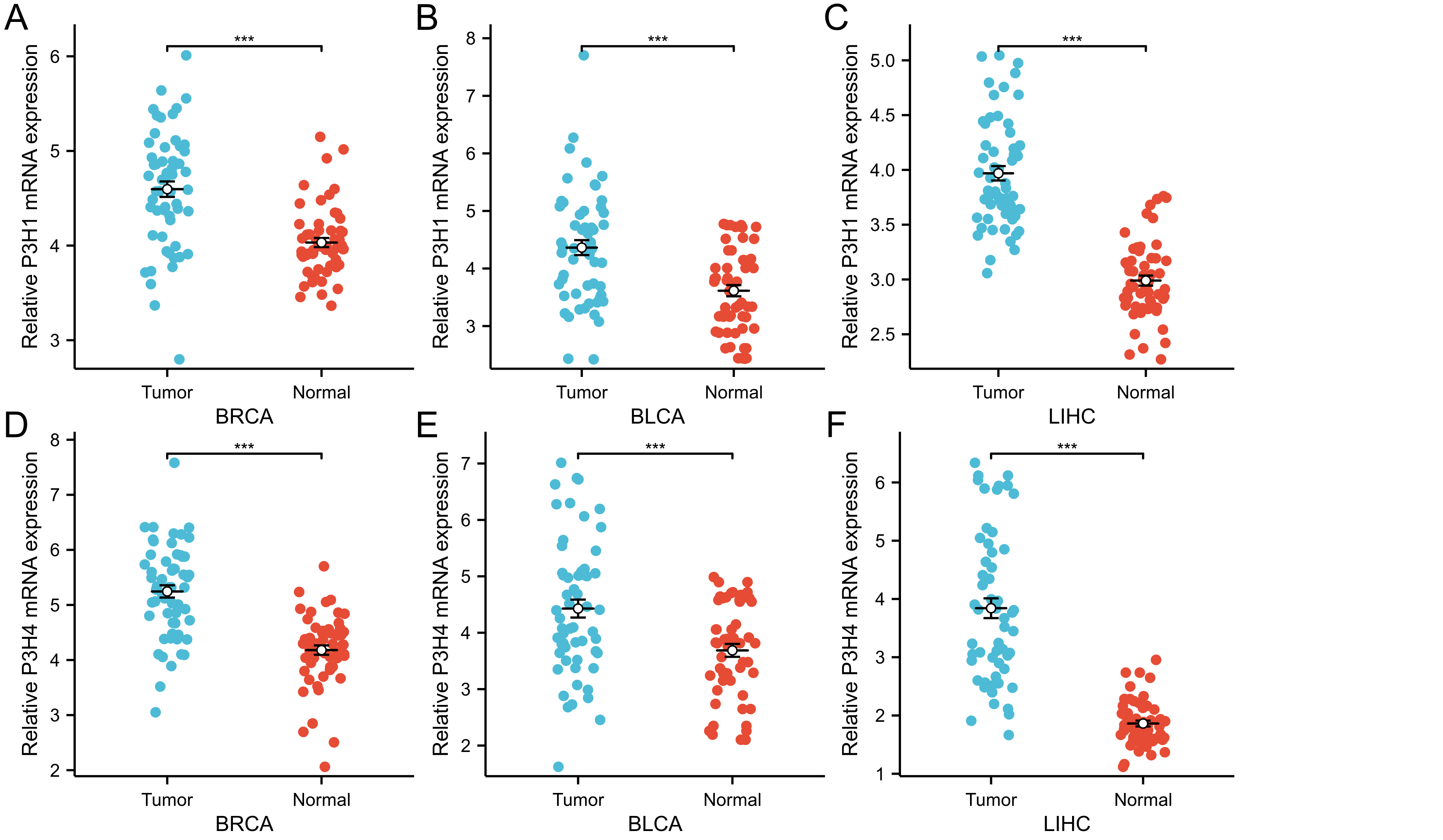

Supplement: Supplementary Figure 1 — Validation of clinical samples. [file Image_1.tiff]
